# Mapping miRNA Research in Schizophrenia: A Scientometric Review

**DOI:** 10.3390/ijms24010436

**Published:** 2022-12-27

**Authors:** Mengyu Lim, Alessandro Carollo, Michelle Jin Yee Neoh, Gianluca Esposito

**Affiliations:** 1 Psychology Program, School of Social Sciences, Nanyang Technological University, Singapore 639818, Singapore; 2 Department of Psychology and Cognitive Science, University of Trento, 38068 Rovereto, Italy

**Keywords:** microRNA, schizophrenia, genetics, scientometry, miRNA, CiteSpace, document co-citation analysis, DCA

## Abstract

Micro RNA (miRNA) research has great implications in uncovering the aetiology of neuropsychiatric conditions due to the role of miRNA in brain development and function. Schizophrenia, a complex yet devastating neuropsychiatric disorder, is one such condition that had been extensively studied in the realm of miRNA. Although a relatively new field of research, this area of study has progressed sufficiently to warrant dozens of reviews summarising findings from past to present. However, as a majority of reviews cannot encapsulate the full body of research, there is still a need to synthesise the diversity of publications made in this area in a systematic but easy-to-understand manner. Therefore, this study adopted bibliometrics and scientometrics, specifically document co-citation analysis (DCA), to review the literature on miRNAs in the context of schizophrenia over the course of history. From a literature search on Scopus, 992 papers were found and analysed with CiteSpace. DCA analysis generated a network of 13 major clusters with different thematic focuses within the subject area. Finally, these clusters are qualitatively discussed. miRNA research has branched into schizophrenia, among other medical and psychiatric conditions, due to previous findings in other forms of non-coding RNA. With the rise of big data, bioinformatics analyses are increasingly common in this field of research. The future of research is projected to rely more heavily on interdisciplinary collaboration. Additionally, it can be expected that there will be more translational studies focusing on the application of these findings to the development of effective treatments.

## 1. Introduction

Approximately thirty years ago, the first instance of micro RNAs (miRNAs) was discovered in the species of *Caenorhabditis elegans* (*C. elegans*) [1]. Sequences of relatively short nucleotides, miRNA are non-coding but conserved RNA sequences that function as regulators of gene expression. As science progressed, it was found during the turn of the century that miRNA were present not only in *C. elegans*, but also in humans [2]. Since then, thousands of unique miRNA sequences have been found in our species. Due to their collective function in regulating the expression of over one-third of the human genome [3], miRNAs serve as compelling molecules that can predict human development at a genomic level, and may serve dual purposes of acting as biomarkers and as therapeutic targets should abnormalities arise.

One such disorder that had garnered the attention of miRNA researchers is schizophrenia. As a complex neuropsychiatric disorder with a strong genetic component (up to 80% hereditability estimates; [4]), the aetiology of schizophrenia is still unclear. Nonetheless, it is thought that with the diversity of miRNAs present in the human genome and their functions in multiple post-transcriptional processes (e.g., stabilising RNAs, up- or down-regulating transcription, RNA translation) [5], together with the high levels of miRNA expression and activity in the brain [6], miRNAs may play a key role in determining the vulnerability of the individual in developing schizophrenia. This may explain the complexity of schizophrenia as a disorder categorised by multiple classes of symptoms: positive (e.g., delusions, hallucinations, disorganised speech, and behaviour) and negative (e.g., alogia, avolition, and anhedonia). Numerous papers have surfaced with empirical findings supporting the role of miRNA in schizophrenia [7,8].

Many reviews have since attempted to summarise research conducted on elucidating the relationship between various miRNA and schizophrenic symptoms, as well as relevant pathways that influence brain development (in terms of neuronal development and signalling, among others). However, due to the varied nature of these studies, reviews in this field are either not systematic (i.e., narrative review only; e.g., [7,9]) or too narrow in scope (i.e., meta-analysis only; e.g., Liu et al. [10]). This is inevitable because narrative reviews may miss out on relevant publications [11], and it is necessary for meta-analyses to make use of homogeneous datasets. There is, therefore, a need for a different approach that can incorporate the full body of research in this field in a systematic manner.

Hence, the current review adopts scientometry, which is a method of measuring scientific production in a field [12,13], to conduct a systematic review of literature related to miRNAs in the context of schizophrenia. Scientometry allows for the systematic and objective evaluation of the literature that avoids the problems of bias in narrative reviews. This study aims to generate a network of prominent research clusters stemming from document co-citation analysis (DCA), a form of scientometric analysis, and to describe each cluster qualitatively, thereby providing young researchers entering this field a structured, easy-to-follow introduction to this topic, its research specialisations [14], as well as highlight influential papers that serve as the bedrock of this research topic.

## 2. Materials and Methods

The search string “TITLE-ABS-KEY((“microRNA*” OR “miRNA*”) AND (schizophren*))”, reflecting the appearance of microRNA and schizophrenia, as well as their related terms, in either the title, abstract, or keywords indexed in publications, was used. A literature search conducted on Scopus on 20 September 2022 revealed 992 results. Scopus was selected over similar platforms (e.g., Web of Science, Medline) as it provides a wider coverage of indexed journals and recent documents [15,16]. To test this assumption, the same document search was conducted on Web of Science as “(TI = (schizophrenia AND (microRNA* OR miRNA*))) OR (AB = (schizophrenia AND (microRNA* OR miRNA*))) OR (AK = (schizophrenia AND (microRNA* OR miRNA*)))” and it resulted in a reduction of 46.86% in the number of identified documents (N = 527 documents) compared to Scopus. The documents identified in Scopus were published between 2005 and 2022 (close to twenty years of research) and themselves reference 90,406 other documents. The *bibliometrix* package for R [17] was initially adopted to conduct bibliometric analysis on the sample of citing articles.

### 2.1. Data Import into CiteSpace

To conduct the scientometric analysis, the 992 publications, together with their 90,406 references, were imported into CiteSpace (version 6.1.R2, 64 bit) in alignment with standard processes [18,19]. All 992 papers and 89,894 references (99.43% of all references) were successfully imported and converted into CiteSpace Web of Science (WoS) format used in further analysis. Although some of the references used by relevant papers were lost, data loss represents only 0.57% of the dataset, well below the acceptable bounds of 1 to 5% for it to be considered negligible [20]. Next, to minimise repetition, CiteSpace’s “Remove Alias” function was also used to remove identically indexed papers and references.

### 2.2. Document Co-Citation Analysis (DCA)

After the importing and conversion steps, a form of scientometric analysis known as DCA was conducted. DCA analyses co-citation frequencies (where co-citation refers to when two or more papers are cited together in the same publication [21]), where higher co-citation frequencies shared by publications mean that these publications are more semantically alike, and therefore may reveal common research trends and thematic domains [22]. Based on the strengths of co-citation relationships within the dataset, CiteSpace DCA generates a network. In the DCA network, nodes represent single publications, and can either be the 992 papers found to be relevant to the research string (from here on referred to as “citing documents”), or the 89,894 references that were used by these papers to advance research in this area (from here on referred to as “cited documents”). Links between nodes represent the strength of co-citation. Based on these links, groups of nodes representing common areas of research are then identified and clustered using the “Clustering” function on CiteSpace.

In DCA network generation, CiteSpace allows the consideration of several node selection criteria in order to create a visually balanced network representative of the dataset. Specifically, these criteria are the G-index, Top N, and Top N%. The G-index is an extension of the more well-known H-index and also measures author citation score [23]. The G-index is calculated by taking the largest number that is equal to the average number of citations of the author’s most cited *g* number of publications [24,25]. The G-index is accompanied by a scaling factor k in CiteSpace to modify the number of nodes included in the final network [20], where larger k values will produce DCA networks with more nodes. The other two criteria, Top N and Top N%, work similarly by selecting the most cited N number or N% of references within a timeframe (from here on referred to as time slice) to be nodes. As in most scientometric studies, the time slice for this review was fixed at one year per slice.

Different configurations of node selection criteria were applied to generate the DCA network. Namely, G-index where k = 15, 25, 50, 75, Top N where N = 50, 75, and Top N% where N = 10 were used. The decision of which generated DCA network to further examine was made through a combination of visual inspection and evaluative metrics (see the next sub-section). The final DCA network selected was then generated based on a G-index with k = 25.

### 2.3. DCA Network Evaluation Metrics

Several metrics are used in the evaluation of generated DCA networks. They can be classified into either structural or temporal measures. Structural metrics include modularity Q, silhouette, and betweenness centrality. Modularity is a measure of the entire network and its divisibility into distinct clusters [26]. The higher its value (from a minimum of 0 to a maximum of 1), the more divisible or distinct the network clusters are [22]. Secondly, the silhouette is a measure of homogeneity within each cluster (i.e., every cluster has its own silhouette measure), where higher values (from a minimum of −1 to a maximum of 1) represent greater cluster homogeneity [27,28]. Thirdly, betweenness centrality is a measure of how well a single node connects two other arbitrary nodes within the network [18,29]. The higher its value, the more well-connected and central a node/publication is within the network [28].

Temporal metrics include citation burstness and sigma, which measure individual nodes’ prominence in the network. Citation burstness is calculated with Kleinberg’s algorithm [30], where higher numbers (with a minimum value of 0) represent greater increases of citations of the node (i.e., the publication) over time. Therefore, high citation burstness scores indicate publications that have received significant scientific attention [31]. Sigma is calculated by using both citation burstness and betweenness centrality values using the formula: (centrality + 1)${}^{burstness}$, where higher values reflect a greater impact of the node on the network and by extension, are suggestive of the publication’s novelty and significance.

**Figure 1:**
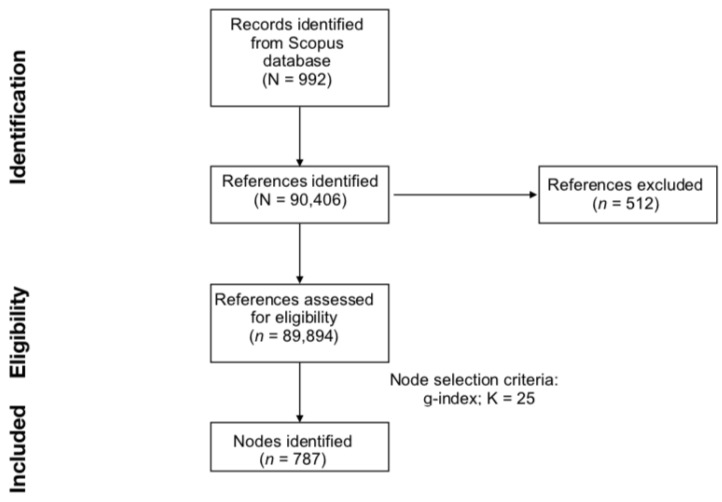
PRISMA flowchart of literature search, document co-citation analysis network generation and evaluation steps.

The literature search, DCA network generation, and evaluation steps are summarised in Figure 1.

## 3. Results

### 3.1. Bibliometric Analysis on the Citing References

**Figure 2:**
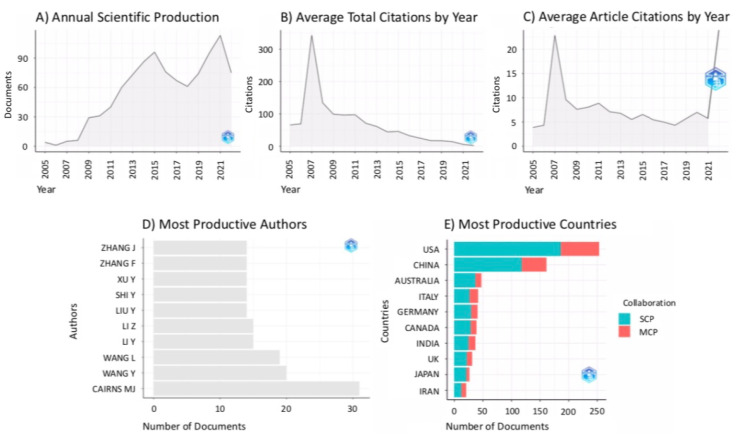
Graphical results of the bibliometric analysis on the citing references by means of the *bibliometrix* package for R [17]. (**A**) Annual scientific production in terms of the number of published documents by year. (**B**) Average total citation obtained by year. (**C**) Average article citations by year. (**D**) Ten most productive authors in the downloaded sample of documents. (**E**) Most productive countries in the downloaded sample of documents. In the plot, the type of collaboration is indicated by colour, with single country publications (SCP) in light blue and multiple country publications (MCP) in red.

From the bibliometric analysis of the citing references, it emerged that the sample of 992 articles developed from 2005 to 2022 with an annual growth rate of 18.82% (see Figure 2A). On average, each document obtained 38.34 citations with an average document citation by year of 4.837 (see Figure 2B,C). The most frequently cited documents were authored by The Schizophrenia Psychiatric Genome-Wide Association Study (GWAS) Consortium [32] (total citations = 1506; total citations per year = 125.5) and by Ingelman-Sundberg et al. [33] (total citations = 924; total citations per year = 57.8).

An total of 9493 keywords associated with Scopus and 2063 keywords selected by the authors indexed the documents. The most popular Scopus-associated keywords were schizophrenia (*n* = 1425 documents), human (*n* = 1194 documents), microRNA (*n* = 1128 documents), humans (*n* = 684 documents), male (*n* = 512 documents), article (*n* = 491 documents), female (*n* = 486 documents), genetics (*n* = 467 documents), microRNAs (*n* = 454 documents), and gene expression (*n* = 432 documents). The most popular author’s keywords were schizophrenia (*n* = 342 documents), microRNA (*n* = 147 documents), miRNA (*n* = 97 documents), bipolar disorder (*n* = 58 documents), epigenetics (*n* = 57 documents), gene expression (*n* = 39 documents), DNA methylation (*n* = 36 documents), microRNAs (*n* = 36 documents), biomarker (*n* = 33 documents), and biomarkers (*n* = 29 documents).

In the data sample, 4338 unique authors were identified. A total of 82 scholars published 93 single-authored documents. On average, the network included 0.229 documents per author and an average of 6.49 co-authors per document. In 30.14% of the cases, documents were published with international co-authorships. The three most productive authors in the data sample were Cairns MJ, Wang Y, and Wang L, with 31, 20, and 19 published documents, respectively (see Figure 2D). The documents’ corresponding authors oftentimes had an affiliation from the United States of America (number of documents = 253; frequency = 0.2808; single country publications (SCP) = 186; multiple country publications (MCP) = 67), China (number of documents = 161; frequency = 0.1787; SCP = 118; MCP = 43), or Australia (documents = 47; frequency = 0.0522; SCP = 37; MCP = 10). In fact, authors with affiliations from the United States of America obtained a total of 15,282 citations with 60.40 average article citations. These authors were followed by the ones having a Chinese (total citations = 3299; average article citations = 20.49) or Australian affiliation (total citations = 2803; average article citations = 59.64) (see Figure 2E).

### 3.2. Document Co-Citation Analysis

**Figure 3:**
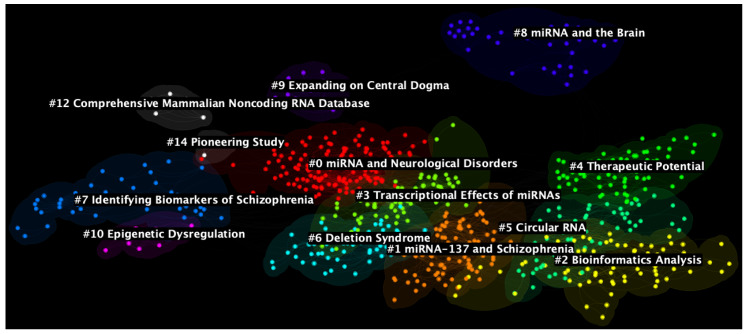
Document co-citation analysis network of all literature on micro RNA and schizophrenia with 13 generated clusters. The image was generated with CiteSpace software [34].

Based on the DCA node selection criteria, a network of 13 clusters, consisting of 787 nodes and 3811 links (i.e., approximately 4.84 links per node), is generated (Figure 3). According to the structural measures, the modularity Q of the network stands at 0.6652, while the average silhouette value of all clusters is 0.8896. From these values and the network diagram, it can be gathered that the clusters are relatively homogeneous, but they are only moderately divisible into separate clusters.

To describe the nature of the clusters, clusters have mean publication years ranging the entirety of the dataset from 2005 to 2020. The three biggest clusters are #0 (mean publication year = 2010), #1 (mean publication year = 2016), and #2 (mean publication year 2020, also the latest mean publication year) with sizes 142, 86, and 80, respectively. The top three most homogeneous clusters are, based on their silhouette scores, #12 (mean publication year = 2005, also the earliest mean publication year), #10 (mean publication year = 2012), and #14 (mean publication year = 2007) with silhouette values = 0.997, 0.995, and 0.991, respectively. Interestingly, these clusters are also the smallest in the whole network. The three earliest clusters are #9 and #12 (mean publication year = 2005), while the three most recent clusters are #2 (mean publication year = 2020), #5 (mean publication year = 2019), and #1 (mean publication year = 2016). For more detailed information on each cluster, please see Table 1.

CiteSpace labels each cluster automatically using the log-likelihood ratio (LLR) algorithm, which generates labels by identifying unique terminologies used in the titles of the contributing papers [18]. However, compared to manual labelling, LLR labels tend to lack accuracy [35] as it lacks the ability to abstract larger themes or concepts from the contributing papers. Where manual labels are more appropriate, they will be suggested in the later discussion by cluster.

Additionally, a total of 136 documents have been identified to have significant citation burstness within the network, where their citation burstness values are all greater than 2.9. For the sake of brevity, only the top 20 documents are represented in Table 2, in accordance with Lim et al. [19], Gaggero et al. [36], Carollo et al. [37]. The significance of these documents’ citation burstness values are determined by gamma, which controls the sensitivity of citation burst detection [16]. In this review, gamma was left at its default value of 1. The top three articles with the highest citation burstness are Bartel [38] (citation burstness = 9.8154), which is a review on microRNAs and highly cited in the years 2009 to 2012 (i.e., duration = 3 years) following its publication in 2004; Pantelis et al. [9] (citation burstness = 9.4755), which is a review by the Schizophrenia Working Group of the Psychiatric Genomics Consortium on genetic loci associated with schizophrenia and highly cited in the years 2016 to 2022 (i.e., duration = 6 years and counting) after its publication in 2014; Perkins et al. [7] (citation burstness = 9.3298) which is the first empirical study to find miRNA associations with schizophrenia and highly cited in the years 2008 to 2014 (i.e., duration = 6 years) immediately after its publication in 2007. In fact, for Pantelis et al. [9] and other papers whose “Burst Ends” are in 2022, it can be assumed that some will continue to enjoy high citations even after this year.

## 4. Discussion

The present paper aims to review research conducted on miRNA and its associations with schizophrenia or schizophrenia-related symptoms. Using scientometric methods, a DCA network of 13 clusters is generated. Additionally, 136 documents with significant citation bursts are detected.

In the subsequent discussion, each cluster will be described qualitatively, in ascending order of their mean year of publication (i.e., oldest to newest). Where their mean publication years are identical, they will be discussed in decreasing cluster size. They will be described in terms of citing papers and cited papers. Citing papers that contribute to each cluster can be characterised by their coverage (number of references in the cluster cited by that paper) and global citing score (GCS: the total number of citations of that paper according to Scopus). References that accompany these citing papers (i.e., cited references) can be characterised by their frequency of citation. Suggested manual cluster labels that replace the LLR labels are also provided, where necessary.

### 4.1. Cluster #9: Expanding on Central Dogma

One of the oldest clusters with its mean publication year in 2005, Cluster #9 comprises the following top citing papers: Perkins et al. [53] (coverage = 14, GCS = 78), Miller and Wahlestedt [54] (coverage = 2, GCS = 149), and Turner et al. [55] (coverage = 2, GCS = 123). As the citing paper with more coverage than the other contributing papers combined, Perkins et al. [53] discusses the importance of non-coding RNA, miRNA included, on the aetiology of neurological disorders. The LLR label of the cluster, central dogma, points to the theory of the flow of genetic information from DNA to RNA and to protein. However, the crux of research into miRNA in fact goes beyond the central dogma to include the regulatory flows of genetic information from DNA to RNA and back to DNA, as non-coding RNA often have a regulatory function in gene expression. As a result, the cited papers in this cluster also reference non-coding RNAs (e.g., Bartel [38]’s landmark review on miRNA with a citation frequency of 23). To better encapsulate the notion of going beyond the theory of central dogma to focus on portions of non-coding RNA in the aetiology of psychiatric disorders, we opt to rename the cluster according to Perkins et al. [53] as “Expanding on Central Dogma”.

### 4.2. Cluster #12: Comprehensive Mammalian Non-Coding RNA Database

The most homogeneous cluster in the network consists of one main citing paper (Pang et al. [56]; coverage = 7, GCS = 130). In Pang et al. [56], a database with over 800 unique non-coding RNAs, including miRNA, was published. The paper paved the way for subsequent studies on publicly available data, and shaping the trend of big data and analyses in the later mentioned cluster. It was also an encouraging early sign of open-source data that enabled replicability analyses [57]. As the cluster LLR label was accurate, no alternative label is suggested to describe the big data approach to study the effects of non-coding RNAs.

### 4.3. Cluster #14: Pioneering Study

Cluster #14, the smallest cluster in the network, consists of one main citing papers (Perkins et al. [7] (coverage = 4, GCS = 431), which was also an influential paper in the overall network for being the first empirical paper to establish a link between miRNA and schizophrenia. This work had been inspired by past studies that uncovered initial evidence that schizophrenia may be due, in part, to post-transcriptional abnormalities through the analysis of messenger RNA (mRNA; [58]). In this paper, 264 different miRNA sequences were examined, of which 16 were found to be expressed differentially in the prefrontal cortex (PFC) of schizophrenia patients as compared to healthy controls [7], laying the groundwork for future miRNA studies in schizophrenia. As the LLR-generated label for this cluster is too broad, the suggested label for this cluster is “Pioneering Study”. The proposed label aims to describe the first studies that found a link between miRNA and schizophrenia.

### 4.4. Cluster #7: Identifying Biomarkers of Schizophrenia

The top three citing papers in this cluster are Straub and Weinberger [59] (coverage = 15, GCS = 69), Tabares-Seisdedos and Rubenstein [60] (coverage = 7, GCS = 173), and Lai et al. [61] (coverage = 6, GCS = 178). A brief survey of citing papers here indicates that the focus is on identifying biomarkers of schizophrenia, particularly from blood samples (e.g., Lai et al. [61] and Woelk et al. [62] with a coverage = 6 and GCS = 22). Blood biomarkers may be a useful and objective diagnostic tool for schizophrenia as brain samples are typically less accessible in living subjects [61,62]. From the above studies, a seven-miRNA signature was uncovered (*hsa-miR-34a*, *miR-449a*, *miR-564*, *miR-432*, *miR-548d*, *miR-572*, and *miR-652*) to be related to negative symptoms of schizophrenia. Additionally, Vawter et al. [63] (coverage = 4, GCS = 16) describes the gene expression quantitative trait loci (eQTL) analytic procedure for identifying biomarkers in general. In the same vein, referenced cited papers also investigated blood biomarkers in schizophrenia (e.g., Glatt et al. [64], Kuzman et al. [65] with citation frequencies = 4 and 3 respectively). To better describe the cluster’s tentatives of findings blood miRNA-related biomarkers in schizophrania, the cluster is renamed “Identifying Biomarkers of Schizophrenia”.

### 4.5. Cluster #8: miRNA and the Brain

Ingelman-Sundberg et al. [33] (coverage = 11, GCS = 913), Perkins and Jeffries [66] (coverage = 11, GCS = 0), and Omahen [67] (coverage = 8, GCS = 7) make up the top three citing papers in this cluster. Citing papers in this cluster largely investigate the action of miRNAs on brain-expressed proteins. For example, Caputo et al. [68] (coverage = 8, GCS = 89) found that the regulation of brain-derived neurotrophic factors are mediated by *miR-26a* and *miR-26b*, signalling a discovery further upstream whose dysfunction may result in abnormal neuronal development and subsequent disorder. On the other hand, Hansen et al. [47] (coverage = 5, GCS = 215) found significant relationships between brain-expressed miRNAs, *mir-206*, and *mir-198*, and schizophrenia. Cited references in this cluster also focus on miRNA expression in the brain (e.g., Perkins et al. [7], Kim et al. [49] with citation frequencies = 13 and 3 respectively). To reflect the cluster’s focus on uncovering the role of miRNAs in brain development and in the aetiology of schizophrenia, the cluster was renamed “miRNA and the Brain”.

### 4.6. Cluster #0: miRNA and Neurological Disorders

Cluster #0 is the largest cluster in the network. Major citing documents in Cluster #0 are Im and Kenny [69] (coverage = 46, GCS = 305), Xu et al. [70] (coverage = 43, GCS = 71), and Sun and Shi [71] (coverage = 43, GCS = 118). Taking these three papers as examples, a large majority of the citing papers in this cluster are reviews of current literature on the relationship between miRNA and neuropsychiatric and neurodevelopmental/neurodegenerative disorders of the central nervous system, using schizophrenia as an example of the research that had been undertaken, in addition to mood disorders, autism, and others. The interest in miRNA in psychiatric disorders stemmed from earlier studies in oncology and other medical conditions [72]. Based on the strong findings in the medical field, genome-wide association studies into various psychiatric conditions too found significant networks between miRNA and the brain. These reviews have summarised the role of miRNAs in brain development and function by regulating gene expression, and where dysregulation or dysfunction of particular miRNAs in the brain are related to developmental differences. In the case of schizophrenia, the role of DiGeorge syndrome critical region gene 8 (DGCR8) *22q11.2* deletion was commonly cited as a well-studied example of miRNA abnormality that is linked to a higher likelihood of developing the disorder. Other miRNAs were also mentioned, largely affecting the development or function of the PFC. For example, *miR-219* is shown to be disrupted in the PFC in schizophrenia. *miR-219* inhibits the signalling of N-methyl-D-aspartate (NMDA) receptors, which may have downstream effects on synaptic action. These citing papers, therefore, made frequent references to empirical papers that investigated the relationship between miRNAs and various disorders. Of these references, the most frequently referenced cited papers in the cluster are Beveridge et al. [73] (frequency = 64) and Stark et al. [74] (frequency = 44). Even though the reviews pertain to a large category of neurological disorders, the evidence stems mainly from research conducted in the realm of schizophrenia, as seen by the commonly referenced cited papers. Due to the broad range of contributing papers in this cluster, where schizophrenia is only one of the examples, an alternative label is suggested: “miRNA and Neurological Disorders”.

### 4.7. Cluster #10: Epigenetic Dysregulation

There are only three citing papers contributing to Cluster #10: Zelena [75] (coverage = 10, GCS = 5), Gavin and Akbarian [76] (coverage = 9, GCS = 28), and Day and Sweatt [77] (coverage = 4, GCS = 74). In addition to miRNA, these review papers discuss epigenetic changes and their contribution to neurological disorders more broadly, including processes such as methylation and histone modification. All epigenetic processes have an effect on cognition [77] and may, therefore, be related to the development of schizophrenia [76]. For example, reduced acetylation at locus *H4K12* in the hippocampus is related to memory impairments (a possible symptom of schizophrenia [78]), which can be improved with subsequent acetylation [77]. As an affirmation of the previous cluster discussion on central dogma and extending beyond, epigenetics represent a consideration of the gene and environment interaction at the molecular level. Therefore, their frequently cited documents are also not necessarily related to miRNA, and may focus instead on other aspects of epigenetic modification. As the cluster revolved around epigenetic mechanisms and their effect on cognition and, possibly, on schizophrenia, the LLR label “Epigenetic Dysregulation” was considered accurate.

### 4.8. Cluster #3: Transcriptional Effects of miRNAs

As the least homogeneous cluster, top citing papers of cluster #3 comprise of Luoni and Riva [79] (coverage = 32, GCS = 33), Merico et al. [80] (coverage = 26, GCS = 34), and Kolshus et al. [81] (coverage = 26, GCS = 34). A reason for its level of homogeneity (or relative lack thereof—its absolute silhouette score is nonetheless still high) can be attributed to several citing papers such as Giridharan et al. [82] that make use of existing literature in the area of miRNA and schizophrenia but instead discuss other disorders (in this case, post-traumatic stress disorder). Nonetheless, of the more relevant citing papers in this cluster, there seems to be a focus on the transcriptional effects of miRNAs on the brain. For example, Millan [83] (coverage = 19, GCS = 159) proposes an epigenetic framework where the regulatory dysfunction of miRNAs contributes to the development of neurological disorders by altering the transcriptional processes of coding genes [84] (coverage = 12, GCS = 120). Likewise, some cited papers in this cluster investigate the miRNA behind dysregulated gene expression, particularly in schizophrenia (e.g., Mellios et al. [85] with a citation frequency = 18, Banigan et al. [86] with a citation frequency = 14, and Smalheiser et al. [87] with a citation frequency = 13). In conclusion, although many documents investigated the role of miRNA in several psychiatric disorders, the cluster included a strong focus on the transcriptional effects of miRNAs on the brain. For this reason, we labelled the cluster “Transcriptional Effects of miRNAs”.

### 4.9. Cluster #6: Deletion Syndrome

Merico et al. [80] (coverage = 21, GCS = 34), Merico et al. [88] (coverage = 21, GCS = 1), and Réthelyi et al. [89] (coverage = 16, GCS = 39) make up the top three citing papers of this cluster. Similar to the papers written by Merico and colleagues [80,88], most citing papers in this cluster focus on the *22q11.2* deletion syndrome and its effect on schizophrenic symptoms (e.g., Merico et al. [90] with a coverage = 6 and GCS = 37 and Forstner et al. [91] with a coverage = 5 and GCS = 40). The *22q11.2* deletion represents the most robustly studied genetic alteration in this network. Merico and colleagues, as the group with the highest number of citing papers in this cluster, conducted a series of studies that found the *22q11.2* deletion increases the likelihood of developing schizophrenia due to its downstream effects in miRNA processing [80,90]. For example, genes targeted by miRNA affected with the *22q11.2* deletion have previously been found to be candidate genes for schizophrenia [80]. In terms of cited references, Karayiorgou et al. [92] (citation frequency = 20), Earls et al. [93] (citation frequency = 9), and Forstner et al. [91] (citation frequency = 7) also study the *22q11.2* deletion syndrome. As several documents studied the link between the *22q11.2* deletion syndrome and its effect on schizophrenic symptoms, the LLR label “Deletion Syndrome” was considered accurate.

### 4.10. Cluster #1: miRNA-137 and Schizophrenia

The main citing documents of the second-largest cluster, cluster #1, include Luoni and Riva [79] (coverage = 40, GCS = 33), Sun and Shi [71] (coverage = 31, GCS = 118), and Kolshus et al. [81] (coverage = 31, GCS = 34). Although the top citing documents here address miRNAs and neurological disorders as a broad category like in the previous cluster, a majority of citing papers focus specifically on schizophrenia only. In fact, the LLR-generated label “induced pluripotent stem cells” (iPSC) indicate a possible source of evidence from where miRNA contribution to schizophrenia aetiology was uncovered. For example, citing papers Hoffmann et al. [94] (coverage = 21, GCS = 14), Ahmad et al. [95] (coverage = 20, GCS = 26), and Liu et al. [96] (coverage = 6, GCS = 14) are reviews of schizophrenia aetiology based on evidence generated by iPSC technology, as iPSC allow the activation of cells that are close to the prenatal state, thereby removing confounds of time and life course changes. This is particularly significant in studies attempting to study the effect of miRNA on prenatal brain development [97] when considering early disease progression and/or heredity. Nonetheless, a more appropriate cluster label is necessary here as there are many other citing papers that did not reference iPSC (e.g., Gibbons et al. [98], Cao and Zhen [99], Wright et al. [100]). For example, Gibbons et al. [98] and Wright et al. [100] examine polymorphisms of miRNA, particularly in *miR-137*. Specifically, polymorphisms of *miR-137* are associated with lower occipital, parietal, and temporal lobe grey matter concentration in individuals with schizophrenia, but no such correlations emerged in typical individuals [100]. Likewise, frequently cited references in this cluster also make use of a broad range of evidence concerning *miRNA-137* and schizophrenia (e.g., Pantelis et al. [9] with a citation frequency of 51, as well as Wright et al. [43], Smrt et al. [101] with citation frequencies of 49 and 37, respectively). As the most of the documents investigated the association between *miR-137* polymorphisms and schizophrenia, the cluster could be renamed “*miRNA-137* and schizophrenia”.

### 4.11. Cluster #4: Therapeutic Potential

The top three citing papers of this cluster belong to Alural et al. [102] (coverage = 25, GCS = 43), Lin and Huang [103] (coverage = 19, GCS = 5), and Gruzdev et al. [104] (coverage = 19, GCS = 30). A common theme in this cluster is the citing papers’ focus on treatment (e.g., Swathy and Banerjee [105] with a coverage = 10, GCS = 39, and Deraredj Nadim et al. [106] with a coverage = 9, GCS = 27). For example, Alural et al. [102] summarises miRNA regulation changes as a result of exposure to different classes of drugs for schizophrenia, and proposes that these miRNA can be targeted specifically in future treatments (e.g., the deleterious effects of dizocilpine on *miR-219*, which were previously discussed in Cluster #0, can be combated with haloperidol and clozapine; [107]). This was agreed on by Deraredj Nadim et al. [106]. Similarly, cited documents focus on changes in miRNA pre- and post-drug exposure (e.g., Song et al. [108] with a citation frequency = 17). The LLR label “Threabeutic Potential” suggests the potential of studying miRNA regulation as a target for future treatment for schizophrenia.

### 4.12. Cluster #5: Circular RNA

The top three citing papers in this cluster were authored by Roy et al. [109] (coverage = 12, GCS = 31), van Calker and Serchov [110] (coverage = 11, GCS = 3), and Shi et al. [111] (coverage = 8, GCS = 8). A brief survey of these citing papers showed that like cluster #3, this cluster focuses on epigenetic effects of miRNA (e.g., Smigielski et al. [112] with a coverage = 7 and GCS = 53; Kuehner et al. [113] with a coverage = 6 and GCS = 69; Gürel et al. [114] with a coverage = 4 and GCS = 11). Therefore, to distinguish this cluster, we look to the cited papers. Frequently cited papers discuss the presence of circular RNA, which regulate the activity of miRNA. According to Hansen et al. [115] (citation frequency = 5), it was found that circular RNA *ciRS-7* and miRNA *miR-7* are highly co-expressed in the brain, which indicated endogenous interaction between the two molecules. This finding was generalised in Memczak et al. [116] (citation frequency = 3) whose review found that circular RNA had the ability to regulate miRNA activity with downstream implications in brain development (e.g., in terms of exon splicing). Within the context of schizophrenia, Mahmoudi et al. [117] (citation frequency = 3) found a lower complexity and depletion of circular RNAs in the dorsolateral PFC of individuals with schizophrenia, which may be linked to dysregulated neuron differentiation and maturation. Therefore, based on the reference to the literature regarding circular RNA and its role as a modulator of miRNA activity, this cluster has been renamed “Circular RNA”.

### 4.13. Cluster #2: Bioinformatics Analysis

The third largest, and also the most recent, cluster has the following top citing papers: van Calker and Serchov [110] (coverage = 14, GCS = 3), Tsermpini et al. [118] (coverage = 11, GCS = 0), and Ghafouri-Fard et al. [119] (coverage = 11, GCS = 6). However, the label from this cluster stems from several citing papers that used a bioinformatics approach (e.g., Sabaie et al. [120] with a coverage = 8 and GCS = 3, Sabaie et al. [121] with a coverage = 7 and GCS = 0, and Jin et al. [122] with a coverage = 5 and GCS = 0). The emergence of this cluster is unsurprising, particularly given its relatively late mean year of publication, as it aligns with the larger research trend of using big data, data mining, and/or other computational methods to derive meaningful patterns [123]. For example, in the two studies by Sabaie et al. [120,121], data mining was used on a large dataset from a national genetic database to identify candidate RNA sequences whose aberrant expressions are involved in the development of schizophrenia. Previous biomarker research was limited due to limited/relatively smaller sampling and differences in analytic methods [124]. With advances in bioinformatics, the integration of multiple datasets is now possible, where more comprehensive, but also more accurate, results can be generated [124]. Owing to the relevance of bioinformatics analysis methods and the big data approach in this cluster, the LLR-generated label is unchanged.

### 4.14. Study Evaluation

There are some limitations of the present review. To examine each in chronological order, we would have to begin with the choice of the search string. Even though the keywords used were intuitive, there may nonetheless still exist publications that are relevant but did not index both miRNA and schizophrenia simultaneously in their title, abstract, or keywords. These entries would be excluded from our analysis [34]. As with any systematic literature search on an indexing database, unpublished papers would also be excluded [125], creating perhaps a skew towards positive results due to publication bias [126]. Conversely, papers that fulfil the search string criteria but only discuss miRNAs and schizophrenia in their periphery would be included, although it may be argued that these papers are still relevant.

Moving to the analysis, it should be noted that DCA is a purely quantitative measure of co-citation frequencies, with the underlying assumption that high co-citation frequencies mean research commonalities and trends [22]. However, based on DCA alone, it is impossible to tell if citing papers agree or disagree with their cited papers [37]. The quality of the paper cannot also be determined solely based on the metrics described above. In fact, DCA ought to be followed with a qualitative discussion, as was done in this review, so that finer nuances can be explored within the generated network [127].

As can be observed, the repeated appearances of top citing papers across many clusters point to both the relevance of their research to the entire cluster, but also to the similarities in thematic focus across clusters. This is expected due to the (relatively) niche research topic to begin with. Therefore, even though distinct cluster labels have been generated and proposed where applicable, the reader should still recall that the clusters are not highly distinct (separate) from each other.

## 5. Conclusions

For a relatively new area of study, a rich body of literature has been established linking the role of miRNA to schizophrenia. Inspired by the previous findings on non-coding RNA and mRNA, miRNA research has branched into schizophrenia among other medical and psychiatric conditions. The gene and environment perspective is emphasised with the introduction of epigenetic frameworks that investigate changes in genetic activity due to external influences. This perspective poses the challenge of understanding which are the external modulators of the genetic expression and how exert their influence, especially in psychiatric conditions such as schizophrenia [128,129]. Finally, with the rise of big data, bioinformatics analyses are increasingly common and are expected to provide new insight into the identification of possible biomarkers for schizophrenia in a genome-wide fashion (e.g., [32,130,131,132]). The future of research in miRNA within the context of schizophrenia can also be expected to rely more heavily on interdisciplinary collaboration (as seen in the latest trends of bioinformatics and big data requiring expertise in biology and computing). Additionally, with the gradual consolidation of the most pertinent miRNAs and regulatory pathways related to schizophrenia, it can be expected that there will be an increase in translational studies focusing on the application of these findings to the development of effective treatment options that take into consideration the individual genetic profile (e.g., [133]). Finally, although biomedical advances show great promise in the understanding and treatment of schizophrenia, psychological perspectives should not be neglected. Future research may also wish to integrate miRNA-based perspectives with psychosocial ones in order to achieve the most comprehensive picture of the disorder, particularly as treatment options ought to be targeted at the individual, rather than the disease.

## Figures and Tables

**Table 1:** Summary statistics for all clusters in final document co-citation analysis network.

Cluster ID	Size	Silhouette	Mean Year	LLR Label	Suggested Label
0	142	0.856	2010	mRNA Gene	miRNA and Neurological Disorders
1	86	0.911	2016	Induced Pluripotent Stem Cell	*miRNA-137* and Schizophrenia
2	80	0.859	2020	Bioinformatics Analysis	Bioinformatics Analysis
3	72	0.845	2013	Psychiatric Disorder	Transcriptional Effects of miRNAs
4	71	0.893	2016	Therapeutic Potential	Therapeutic Potential
5	63	0.898	2019	Exploiting Circulating Micro RNA	Circular RNA
6	59	0.878	2013	Deletion Syndrome	Deletion Syndrome
7	45	0.909	2008	Schizophrenia Gene	Identifying Biomarkers of Schizophrenia
8	38	0.988	2008	Clinical Aspect	miRNA and the Brain
9	16	0.99	2005	Central Dogma	Expanding on Central Dogma
10	10	0.995	2012	Epigenetic Dysregulation	Epigenetic Dysregulation
12	7	0.997	2005	Comprehensive Mammalian Noncoding RNA Database	Comprehensive Mammalian Noncoding RNA Database
14	4	0.991	2007	Schizophrenia	Pioneering Study

**Table 2:** Top 20 unique documents with highest citation bursts. Statistics are rounded to two decimal places.

Reference	Citation Burstness	Publication Year	Burst Begin	Burst End	Duration	Betweenness Centrality	Sigma
Bartel [38]	9.82	2004	2009	2012	3	0.08	2.09
Pantelis et al. [9]	9.48	2014	2016	2022	6	0.02	1.24
Perkins et al. [7]	9.33	2007	2008	2014	6	0.04	1.46
Filipowicz et al. [39]	8.90	2008	2009	2010	1	0.01	1.07
Abelson et al. [40]	8.55	2005	2007	2010	3	0.07	1.81
Ripke et al. [41]	8.53	2013	2015	2019	4	0.02	1.17
Schratt et al. [42]	8.40	2006	2007	2014	7	0.03	1.29
Siegert et al. [8]	8.00	2015	2016	2018	2	0.02	1.20
Wright et al. [43]	7.98	2013	2014	2018	4	0.01	1.05
Lewis et al. [44]	7.61	2005	2007	2011	4	0.08	1.80
Barry et al. [45]	7.15	2014	2018	2022	4	0.02	1.15
Shi et al. [46]	6.72	2012	2014	2016	2	0.00	1.02
Hansen et al. [47]	6.51	2007	2009	2012	3	0.03	1.19
The Schizophrenia Psychiatric GWAS Consortium [32]	6.43	2011	2014	2018	4	0.04	1.30
Sun et al. [48]	6.36	2011	2014	2016	2	0.00	1.00
Kim et al. [49]	6.21	2007	2008	2013	5	0.04	1.25
Gardiner et al. [50]	6.14	2012	2014	2017	3	0.01	1.04
Zhou et al. [51]	6.00	2009	2010	2013	3	0.02	1.12
Guan et al. [52]	5.98	2014	2015	2018	3	0.02	1.12
Beveridge and Cairns [5]	5.95	2012	2016	2018	2	0.00	1.01

## Data Availability

Not applicable.

## Abbreviations

The following abbreviations are used in this manuscript:

miRNA	Micro Ribonucleic Acid
DCA	Document Co-Citation Analysis
WoS	Web of Science
PRISMA	Preferred Reporting Items for Systematic Reviews and Meta-Analyses
GWAS	Genome-Wide Association Study
SCP	Single Country Publications
MCP	Multiple Country Publications
LLR	Log-Likelihood Ratio
GCS	Global Citing Score
mRNA	Messenger RNA
PFC	Prefrontal Cortex
eQTL	Gene Expression Quantitative Trait Loci
DGCR8	DiGeorge Syndrome Critical Region Gene 8
NMDA	N-Methyl-D-Aspartate
iPSC	Induced Pluripotent Stem Cells
